# ZnO size and shape effect on antibacterial activity and cytotoxicity profile

**DOI:** 10.1038/s41598-022-12134-3

**Published:** 2022-05-17

**Authors:** Nataliya Babayevska, Łucja Przysiecka, Igor Iatsunskyi, Grzegorz Nowaczyk, Marcin Jarek, Ewa Janiszewska, Stefan Jurga

**Affiliations:** 1 NanoBioMedical Centre, Adam Mickiewicz University in Poznan, Wszechnicy Piastowskiej 3, 61-614 Poznań, Poland; 2 Faculty of Chemistry, Adam Mickiewicz University in Poznan, Uniwersytetu Poznanskiego 8, 61-614 Poznań, Poland

**Keywords:** Cell biology, Microbiology, Materials science, Nanoscience and technology, Optics and photonics

## Abstract

The aim of our work was the synthesis of ZnO nano- and microparticles and to study the effect of shapes and sizes on cytotoxicity towards normal and cancer cells and antibacterial activity toward two kinds of bacteria. We fabricated ZnO nano- and microparticles through facile chemical and physical routes. The crystal structure, morphology, textural properties, and photoluminescent properties were characterized by powder X-ray diffraction, electron microscopies, nitrogen adsorption/desorption measurements, and photoluminescence spectroscopy. The obtained ZnO structures were highly crystalline and monodispersed with intensive green emission. ZnO NPs and NRs showed the strongest antibacterial activity against *Escherichia coli* and *Staphylococcus aureus* compared to microparticles due to their high specific surface area. However, the ZnO HSs at higher concentrations also strongly inhibited bacterial growth. *S. aureus* strain was more sensitive to ZnO particles than the *E. coli.* ZnO NPs and NRs were more harmful to cancer cell lines than to normal ones at the same concentration.

## Introduction

Successful fabrication of materials with desired properties using simple and low-cost approaches attracts a great deal of attention. Materials properties are strongly dependent on their characteristics, mainly phase composition, crystallinity, shape and size, dispersion, etc. Materials morphology plays an important role in optics, photonics, sensors, and solar cells to obtain high-quality devices^1–3^. For example, photocatalytic activity of semiconductor materials (*e.g.* NiO, TiO_2_) depends strongly on their particle sizes, basically due to enhancing specific surface area^4,5^.

Materials for bioapplication, such as biomarkers, biosensors, or drug carriers with improved optical, sensing, and surface properties can be obtained due to changing their shape, size, and dimension^6,7^. Nanoparticles due to their small size have exciting physicochemical properties, such as a high specific surface-to-volume ratio, a size similar to the size of biomolecules allowing for their greater mobility and passing through the cell biological membranes^8^. Morphology and specific surface area affect the antibacterial activity, therefore, it is necessary to synthesize a material with a novel morphology having a higher amount of adsorption sites and surface area^9–11^. Thus, verifying the morphology of the materials and hence their specific surface area can show great potential for bioapplication because the properties can be tuned and improved.

Decreasing the particle size (nanosized materials, such as 0D materials (*e.g.* quantum dots, QDs)) is one of the effective ways to increase the surface area. Despite many advantages of the nanoparticles, often their main disadvantage is particle agglomeration. Modern approaches allow obtaining high-quality nanoparticles without any stabilizers and surfactants^12,13^. Anisotropic multidimensional and multicompartmental particles with intriguing morphologies are the subjects of extensive research. One dimension materials (1D) offer high specific surface area because of their unique morphologies. 3D materials are seen to be more suitable for many applications than other morphologies/structures as they may be assembled from lower dimensional materials and provide a large specific surface area and fast electron transport^14^.

Metal oxide nanoparticles have great attention in materials science and nanotechnology for a large variety of applications. From semiconductor materials, zinc oxide (ZnO) is an important multifunctional material with broad applications in many scientific fields. As a promising semiconductor oxide (large exciton binding energy (60 meV and wide bandgap Eg = 3.37 eV) ZnO possess excellent physicochemical properties, such as high thermal and chemical stabilities, and thermal conductivity, UV protection or electronic, photonic, unique optical, and luminescent properties. At the same time, ZnO is a low-cost material, its synthesis is simple and it can be obtained with a big variety of morphology using physical and chemical approaches. ZnO with different morphology and dimension (*e.g.* bulk ZnO, nanoparticles, films, 3D hierarchical structures) has broad applications as sensors, catalysts, solar cells, and optoelectronic devices^15–17^. Furthermore, zinc is a necessary element of our health and has high biocompatibility with human cells, low toxicity, and good antimicrobial activity^10,18,19^. All of these properties allow using ZnO materials in medicine as biomarkers, drug carriers, and therapeutic^20,21^. Thus, Warheit et al.^22^ studied the effect of ZnO morphology on the toxicity of lung cells. They showed a low cytotoxic effect on rat L2 lung epithelial cells and primary rat lung alveolar macrophage treated with ZnO nanoparticles (NPs). Li et al.^23^ studied the cell viability of ZnO nanowires using two cell lines. Results showed that ZnO nanowires were biocompatible to HeLa cells up to the highest concentration used (up to 100 μg/mL), whereas for L929 cells they were deleterious even at lower concentrations.

ZnO possesses unique antibacterial, antimicrobial and antifungal properties, ZnO-based materials can initiate antibacterial activity even without the presence of light^24^. Like other metal oxides, zinc oxide can change its physicochemical characteristics under the influence of the environment, and, unfortunately depending on its concentration, ZnO can be hazardous to the life of living organisms and ecosystem safety. Therefore, special attention is paid to the control of the release of toxic zinc ions from the solution, especially in vivo studies^25,26^. Lee et al.^27^ showed that the toxicity of ZnO NPs can be correlated well with the zinc concentration and through transformation thought interaction ZnO with sulphur, phosphate, and ferric oxide. The maximum toxicity reduction was observed after phosphation, attributable to the inhibition of zinc release by phosphates rapidly enclosing ZnO NPs.

In presented studies^19,28,29^, authors obtained ZnO with different morphology and indicated that ZnO and its composites can be used as good antibacterial agents toward Gram-positive, as well as Gram-negative bacteria. Thus, despite intensive research in this field, the evaluation of ZnO antibacterial activity and biocompatibility due to changing the particle morphology is still important and can significantly expand the scope of this material in biomedical applications.

The main aim of our work was a synthesis of ZnO nano- and microparticles, the study of their crystal structures, and the effect of morphology and specific surface area on luminescent properties, cytotoxicity, and antibacterial activity toward Gram-positive and Gram-negative bacteria.

## Results

### Fabrication of ZnO nano- and microparticles

**Figure 1:**
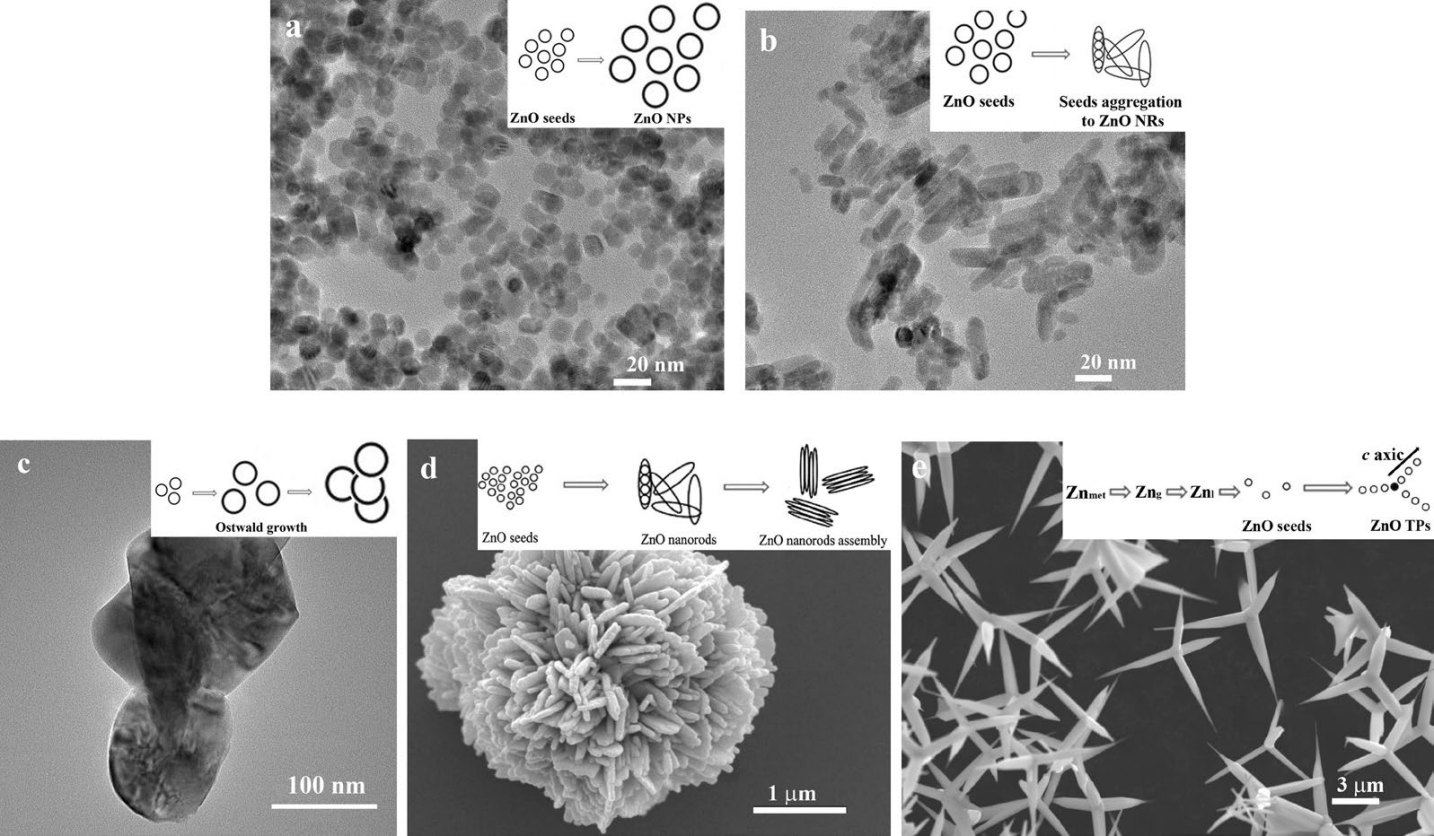
TEM and SEM images of ZnO nano- and microparticles: nanoparticles (**a**), nanorods (**b**), particles (**c**), hierarchical structures (**d**), tetrapods (**e**).

ZnO nano- and microparticles with different morphology were fabricated using facile chemical and physical methods. We performed the study of the shape and size of the obtained ZnO nano- and microparticles using the transmission (HRTEM) and scanning electron microscopes (SEM). Indeed, in nanoparticles synthesis, we obtained the highly crystalline and monodispersed ZnO nanoparticles (ZnO NPs) and nanorods (ZnO NRs) using the sol–gel approach without any additional surfactants or ligands. ZnO NPs had a perfect spherical shape and uniform size distribution with a mean nanoparticle size of approximately 7 nm (Figs. 1a, S1a). The FFT digital diffractogram pattern presented in Fig. S1b from nanoparticles with [010] zone axis orientation, demonstrated individual point reflections that indicate a single crystalline structure of particles. The interplanar distance between adjacent lattice fringes is 2.57 Å corresponding to d(002).

Changing the synthesis parameters in ZnO NP synthesis (reagent concentration, synthesis time, and temperature) we obtained ZnO NRs and ZnO particles (ZnO Ps), (Fig. 1b,c). The mean size of ZnO NRs was approximately 5 × 17 nm (width × length), Fig. S1c. After annealing of ZnO NPs at 900 °C for 2 h, we obtained ZnO Ps with sizes of ~ 150 nm (Fig. 1c).

To obtain ZnO with the hierarchical structure (ZnO HSs) we used a solvothermal reaction. The SEM image in Fig. 1d confirmed the formation of the flower-like (peony) structure of ZnO. All individual particles had a spherical shape, were highly crystalline, and monodispersed with sizes of approximately 4 µm and the thickness of the individual plates of about 100 nm (Fig. S1d).

We also obtained ZnO in the shape of tetrapods using thermal evaporation in the air. ZnO tetrapods (ZnO TPs) were well faceted with four uniform legs connected to a central nucleus (Figs. 1e, S1e). The legs had hexagonal morphology with a length of about 5.2 μm and approximately 170 nm in thickness.

### Structural characterization of ZnO nano- and microparticles

**Figure 2:**
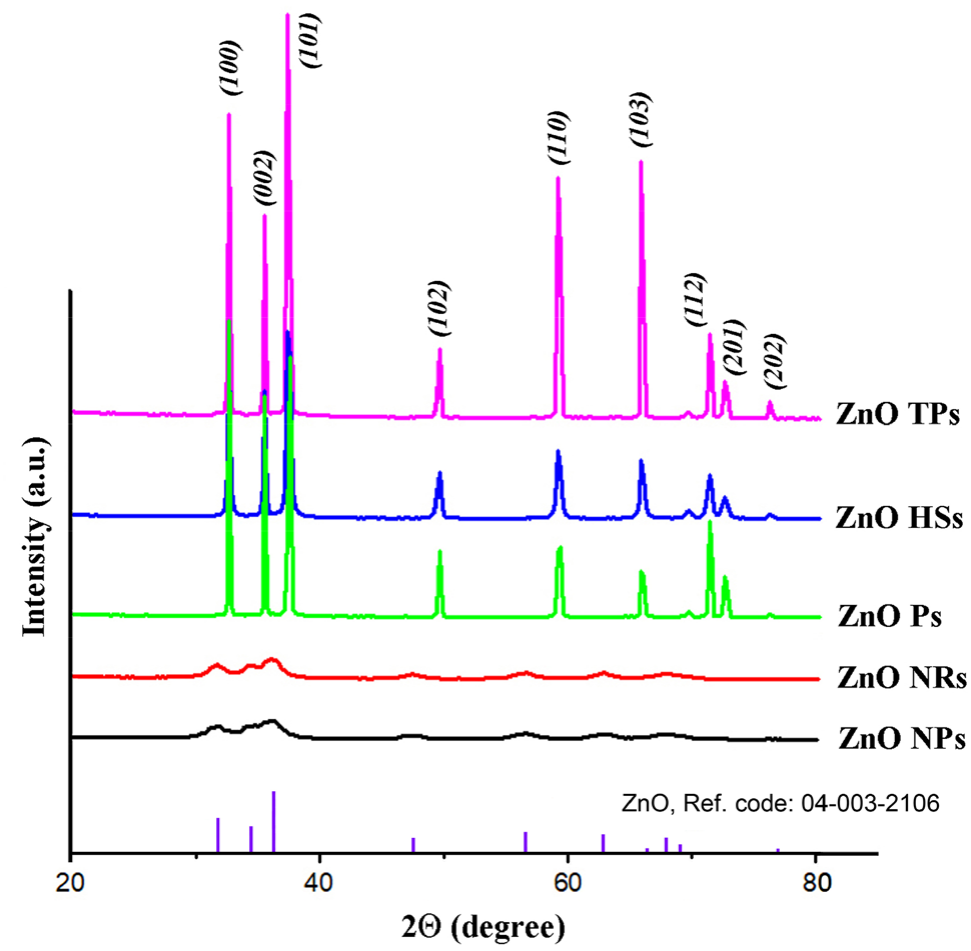
Typical XRD pattern of ZnO nano- and microparticles.

To determine the crystal structure and phase purity of the obtained ZnO materials XRD measurements were performed. Figure 2 shows the XRD pattern of ZnO particles with different morphology. X-ray powder diffraction confirmed the formation of a single-phase of ZnO for all samples.

All diffraction peaks are indexed according to the hexagonal phase of ZnO wurtzite crystal structure with main (100), (002), (101), (102), (110), (103), (112), and small (201) and (202) crystal planes. No additional peaks indicating the presence of impurity phases were found in all studied samples. The broadening of the peaks for ZnO NPs and ZnO NRs samples (Fig. 2, black and red lines) can be attributed to the small particle size of ZnO. Calculated crystallographic parameters of zinc oxide particles are presented in Table 1.

**Table 1:** Crystallographic parameters of the obtained ZnO nano- and microstructures.

Sample	XRD		
	Cell parameters		
	*a*, nm	*c*, nm	*V*, nm^3^
ZnO NPs	0.324	0.526	4.811
ZnO NRs	0.326	0.528	4.853
ZnO Ps	0.324	0.520	4.753
ZnO HSs	0.324	0.521	4.766
ZnO TPs	0.325	0.521	4.764

The results indicate higher lattice constant values for ZnO NPs and ZnO NRs samples compared with the others. The lowest lattice constants *a* and *c* are observed for ZnO Ps and are the results of their preparation condition (high-temperature annealing). Similar results were presented by Lupan et al*.*^30^ indicating a decrease of the lattice constants of ZnO with the increase of the temperature of its treatment.

### Textural properties of ZnO nano- and microparticles

**Figure 3:**
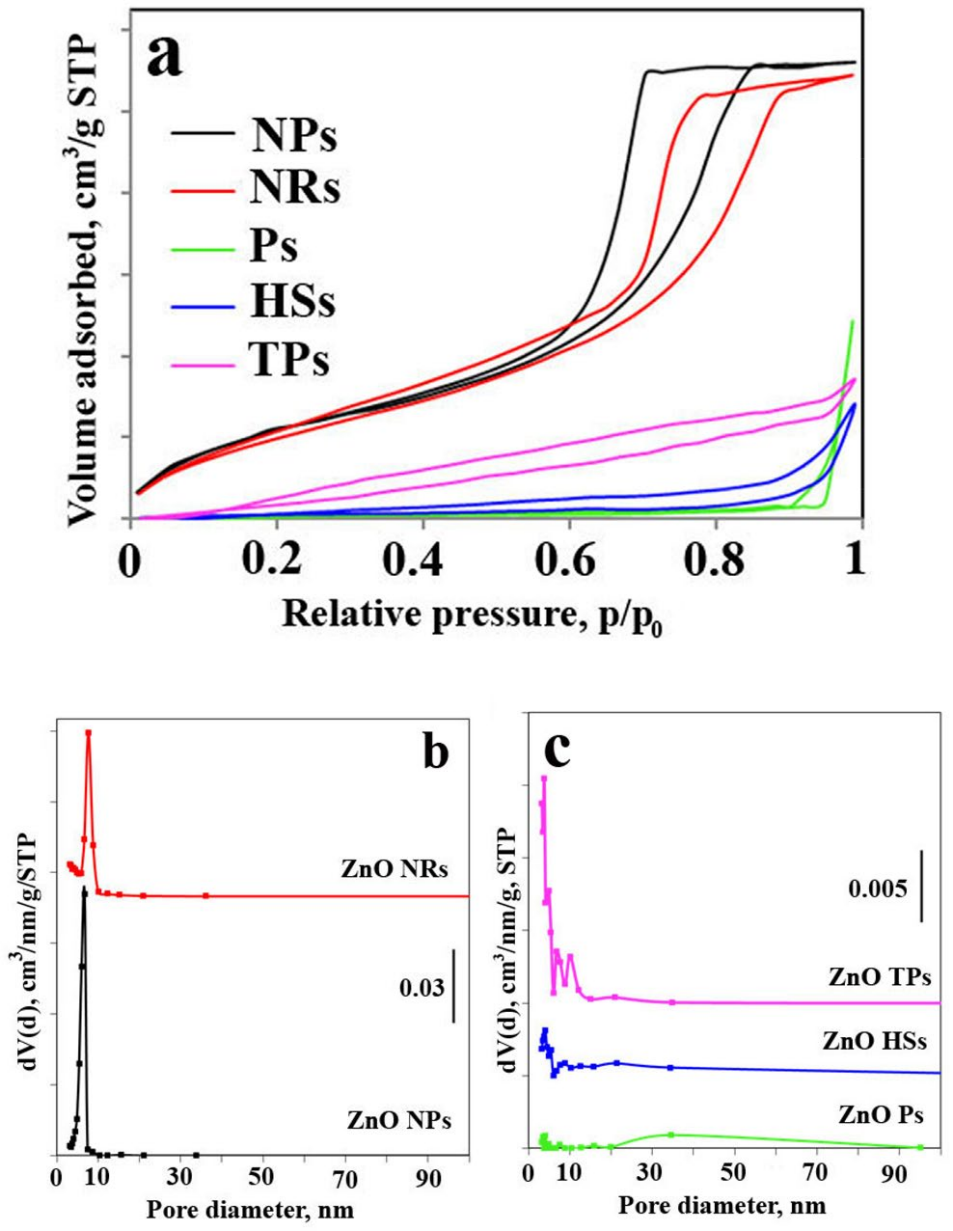
Nitrogen physisorption isotherms and pore size distribution for indicated samples.

The textural properties of the investigated samples were performed by N_2_ adsorption–desorption analysis on powdered ZnO samples at −196 °C. The specific surface area, pore volume, and pore size distribution of samples were specified. The results are summarized in Table 2 and presented in Fig. 3. The adsorption/desorption isotherms of investigated ZnO materials correspond to type IV typical for mesoporous materials. The estimated BET specific surface area of the obtained ZnO nano- and microparticles were 83.5 m^2^/g (NPs), 83.8 m^2^/g (NRs), 3.0 m^2^/g (Ps), 4.5 m^2^/g (HSs), and 29.4 m^2^/g (TPs). The lowest specific surface area, as well as pore volume, showed ZnO Ps and ZnO HSs. The samples with the highest specific surface areas (ZnO NPs and ZnO NRs) showed narrow pore size distribution with most of the pores in the size range from 3 to 10 nm, whereas the samples with lower specific surface areas possess additionally pores with a diameter above 10 nm.

**Table 2:** Surface area and pore volume of ZnO samples with different morphology.

Samples	S_BET_, m^2^/g	Pore volume, cm^3^/g
*ZnO NPs*	83.5	0.18
*ZnO NRs*	83.8	0.17
*ZnO Ps*	3.0	0.08
*ZnO HSs*	4.5	0.04
*ZnO TPs*	29.4	0.04

### Photoluminescence properties of ZnO nano- and microparticles

**Figure 4:**
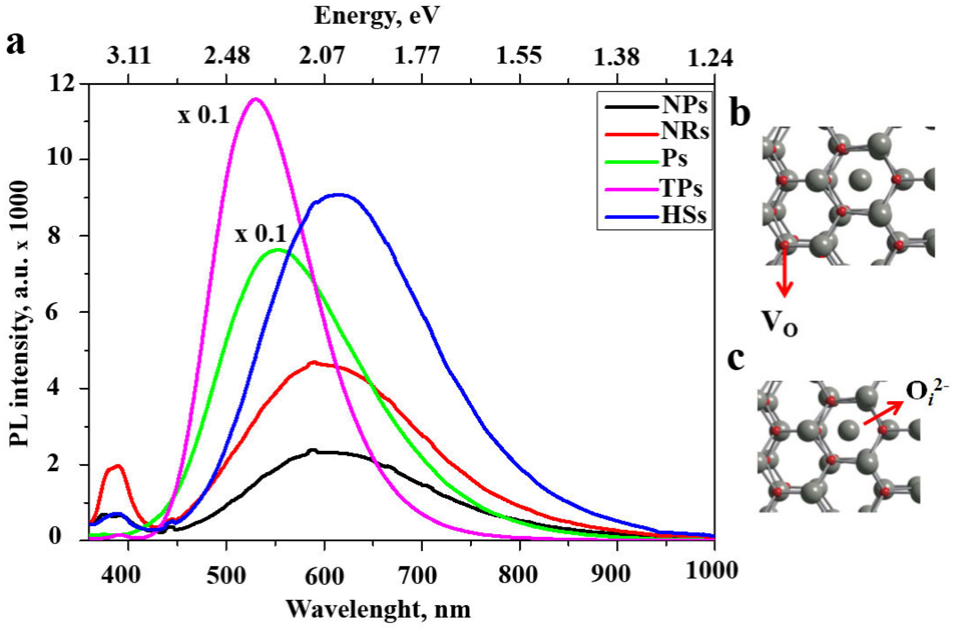
PL spectra of ZnO nano- and microparticles.

The PL spectra of ZnO nanostructures are shown in Fig. 4a. All investigated ZnO nanostructures demonstrate the intense and broad peak in the visible range. The intense emission for ZnO TPs and Ps is mostly associated with oxygen vacancies (Fig. 4b), whereas the PL for ZnO NPs, NRs, and HSs is attributed to oxygen interstitials (Fig. 4c).

### Antibacterial properties of ZnO nano- and microparticles

**Figure 5:**
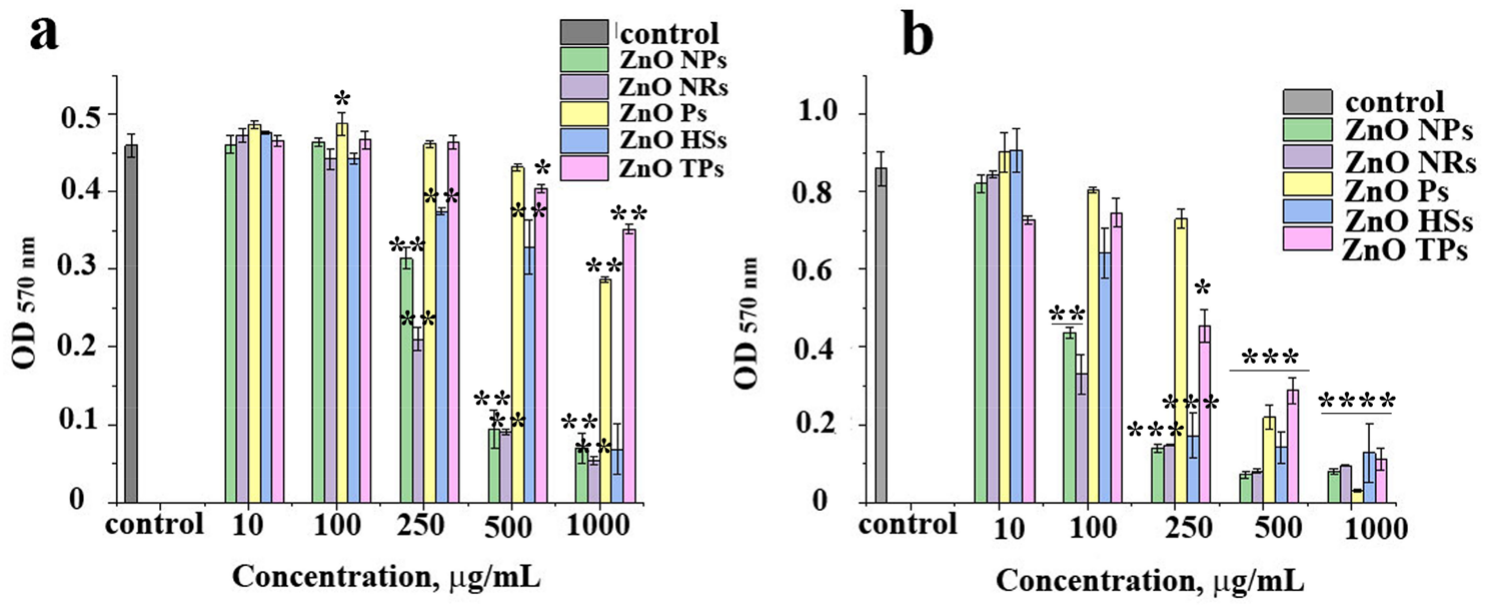
Antimicrobial activity of ZnO nano- and microparticles against *E. coli* (**a**), Asterisks denotes the statistical significant difference compared with controls *p ≤ 0.001, **p ≤ 0.0001; and *S. aureus* (**b**), Asterisks denotes the statistical significant difference compared with controls *p ≤ 0.05, **p ≤ 0.01, ***p ≤ 0.001, ****p ≤ 0.0001, evaluated by measuring optical density at 570 nm (OD_570 nm_) after incubation at 37 °C, 220 rpm for 24 h. Data presented as mean ± SD of three independent experiments, each performed in triplicates.

As a next important step, to use our ZnO-based materials as potential antibacterial agents, the antimicrobial activity of ZnO nano- and microstructures was evaluated against Gram-negative *Escherichia coli* and Gram-positive *Staphylococcus aureus* bacterial strains using optical density measurements after 24-h incubation, as summarized in Fig. 5. By measuring the optical density at 570 nm (OD_570_), the growth of bacteria can be quantified, based on turbidity resulting from light scattering^31^. For all samples, the bacteria number decreased in a concentration-dependent manner. The most effective towards *E. coli* bacterial strains were ZnO nanoparticles and nanorods, as well as ZnO hierarchical structures at the highest concentration, and the least antibacterial effect was observed for ZnO Ps and TPs. The Gram-positive *S. aureus* bacteria strains were more sensitive to the antimicrobial action of ZnO nano- and microparticles than *E. coli*. However, also here, the greatest lowering of the bacteria number was visible after incubation with ZnO NPs, NRs, and HSs with the highest specific surface areas.

### ROS generation assay after ZnO nano- and microparticles treatment

**Figure 6:**
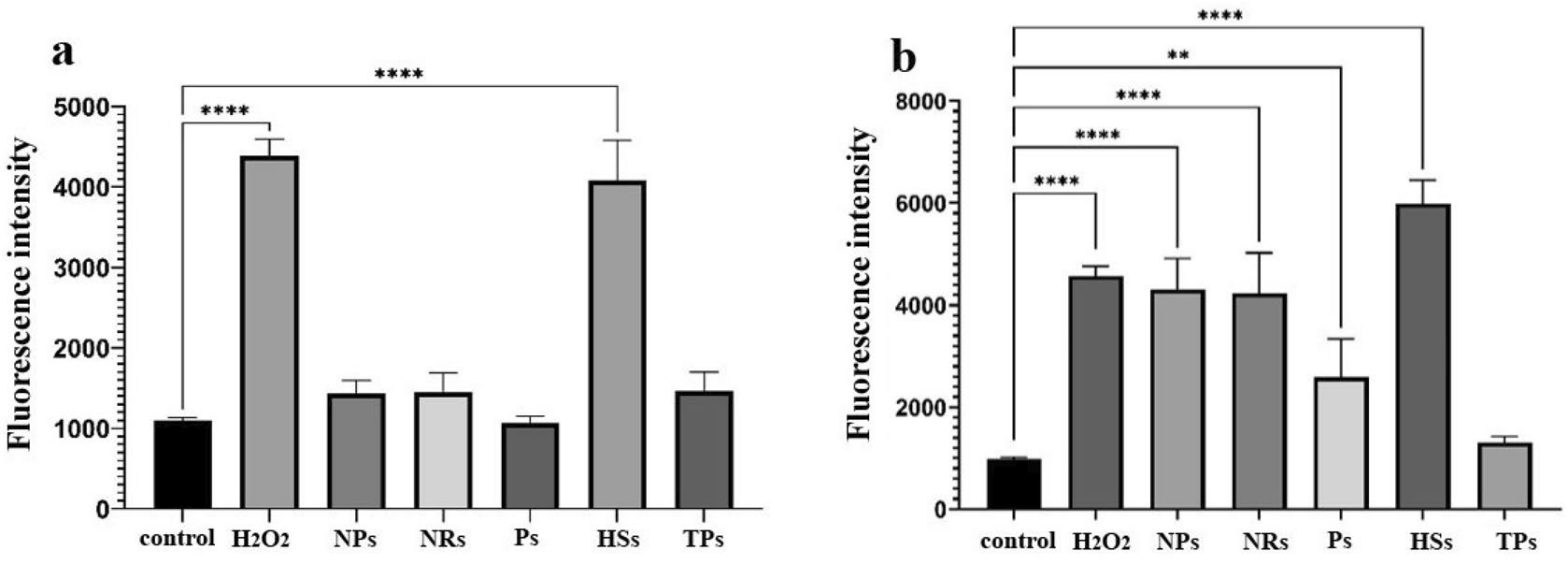
Quantitative evaluation of reactive oxygen species (ROS) generation in *E. coli* (**a**) and *S. aureus* (**b**) bacterial cells using DCFH-DA assay. Results presented as a DCF fluorescence intensity of ROS generation after cells incubation with 100 ug/ml ZnO solutions, compared to non-treated and H_2_O_2_ controls. Asterisks denotes the statistical significant difference compared with controls **p ≤ 0.01, ***p ≤ 0.001, ****p ≤ 0.0001.

Oxidative stress is one of the proposed antibacterial mechanisms of metal nanoparticles^32,33^. The involvement of oxidative stress in the antibacterial activity of ZnO NPs was examined through the measurement of intracellular reactive oxygen species (ROS) production by DCFH-DA assay. In the presence of ROS, non-fluorescent DCFH-DA is oxidized and switches to green-fluorescent dichlorofluorescein (DCF). For this study, both *E. coli* and *S. aureus* bacterial cells were treated with 100 µg/mL of ZnO solutions, whereas H_2_O_2_ was chosen as the positive control and the untreated cells as the negative control. As shown in Fig. 6, in *E. coli* bacteria the weak ROS is generated in control cells, as well as in almost all ZnO nano- and micro-particles-treated cells. A significant increase in fluorescence signal intensity was observed for ZnO heterostructures and H_2_O_2_-treated cells.

The obtained results for *S. aureus* strains were different. Here, only ZnO TPs generate ROS at a similar level as control. The highest increase of DCF fluorescence was for ZnO HSs, but then for NPs and NRs. Even for ZnO Ps, the observed signal was increased. These results are in agreement with bacterial viability assay, thus *S. aureus* was more susceptible to NPs treatment than *E. coli* strain.

### Cytotoxicity studies of ZnO nano- and microparticles

**Figure 7:**
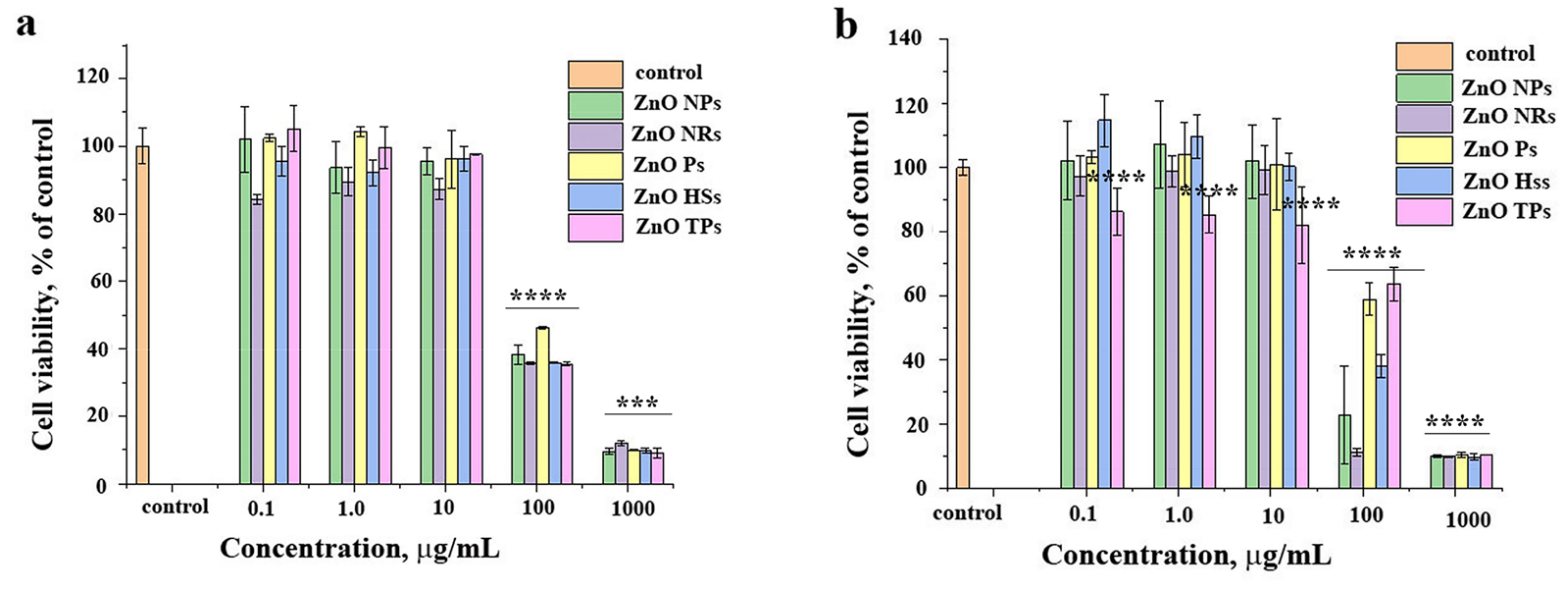
WST-1 assay performed on normal human fibroblasts MSU1.1 (**a**) and cervical cancer HeLa cells (**b**) after 24 h of incubation with indicated ZnO materials, asterisks denotes the statistical significant difference compared with controls ***p ≤ 0.001, ****p ≤ 0.0001.

The impact of distinct ZnO nano- and microparticles on human cells was evaluated by cytotoxicity analysis by WST-1 assay, performed on cervical cancer cell line (HeLa) and normal human fibroblasts (MSU1.1) (Fig. 7). Five different ZnO nano- and microparticles concentrations of 0.1, 1.0, 10, 100, and 1000 µg/mL were employed, and the cell viability was determined after 24 h of treatment. The cell viability data revealed that for both types of cells at the concentration up to 10 µg/mL all kinds of ZnO particles were biocompatible, maintaining a high level of viability (up to 80%). However, at a higher concentration range (100–1000 µg/mL) the cell viability declines to 40%, indicating a high level of toxicity. Interestingly, at a concentration of 100 µg/mL, a similar level of viability was observed for all samples on MSU1.1 cells, while for HeLa cells differences between distinct ZnO solutions were visible, indicating ZnO Ps and TPs less deleterious to HeLa cells.

### Zn ions release studies

**Figure 8:**
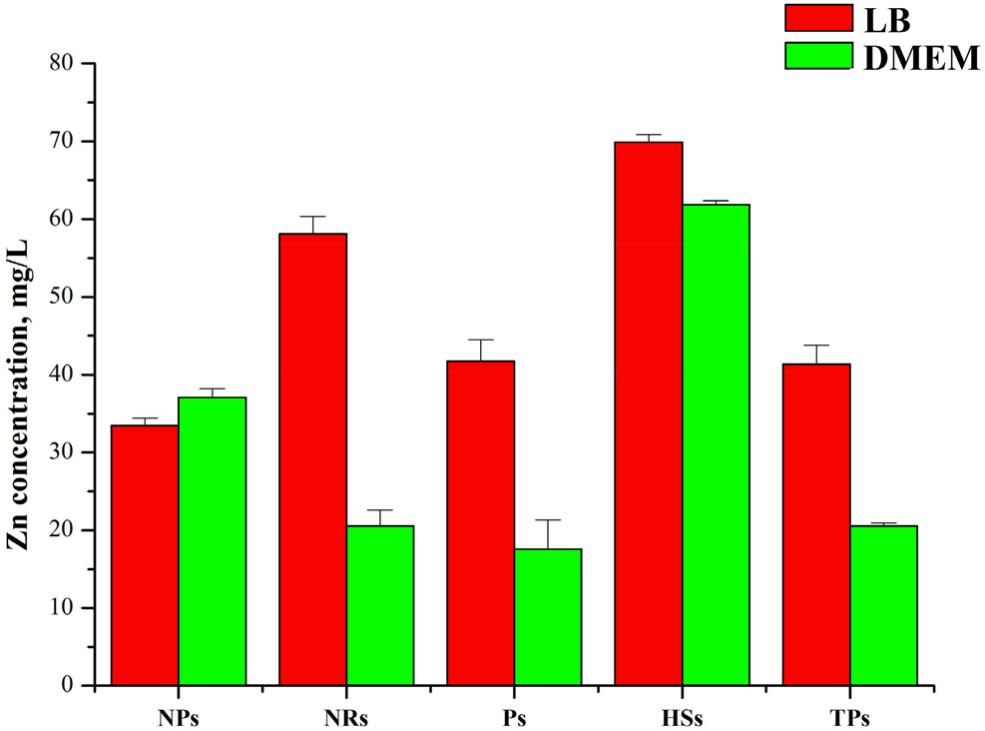
Released zinc ions in DMEM and LB at 24 h.

To study the effect of zinc ions on toxicity, we performed experiments, which determine the release of Zn ions into the bulk solutions (DMEM and LB media). Experiments were performed at 15 min, 30 min, 1 h, 5, and 24 h. All of our studied ZnO nano- and microparticles were released from the pristine ZnO into two selected media (Figs. 8, and S3). Figure 8 shows the concentration of Zn ions in media after 24 h. Results showed that the Zn ion release is linear with increase the time, but is not size-dependent. The maximum amount of released Zn ions in both media (~ 70 mg/L (DMEM) and ~ 62 mg/L (LB) was observed for ZnO HSs. The amount of Zn ions released into LB from NPs, Ps, and TPs was ~ 40 mg/L. In DMEM medium, the minimum Zn ions were released from ZnO Ps.

## Discussion

It is known that materials with different morphology can be obtained due to changing the synthesis parameters. For the synthesis of ZnO nano- and microparticles we used chemical and physical approaches. All obtained ZnO nano- and microparticles were highly crystalline. It is well known that particles in nanorange or materials with unique structures (including hierarchical structure) possess high specific surface area. Often the specific surface area is strongly related to the particle size and increased with decreasing particle size^34^. These results are consistent with the results of particle size estimated by SEM/TEM analysis. The samples with the highest specific surface areas (ZnO NPs and ZnO NRs) showed particles with a size below 10 nm, whereas the samples with lower specific surface areas possess much bigger particles (Fig. 1).

Analysis of PL spectra for different semiconductor nanostructures is a powerful tool to investigate their morphology features, defects, and even chemical composition^35^. Typical ZnO nanostructures, such as nanoparticles, nanorods, etc., exhibit two luminescence bands located in the UV region (the near band emission—NBE) and a broad long-wavelength band at the Visible spectrum (the deep-level emission—DLE)^35^. The unique optical properties are very important for medical and biological visualization applications^36^. Depending on the PL intensity, PL peaks position, and the ratio I_NBE_/I_DLE_, one can conclude about the structural features of produced ZnO nanostructures. It is well known that the DLE is associated with different ZnO defects, such as zinc vacancies (Zn_i_ ^++^), single (V_o_^+^), and double (V_o_^++^) ionized oxygen vacancies, neutral oxygen vacancies (V_o_), and oxygen interstitials (O_i_). According to previous studies, there are three main defects involved in the DLE: V_o_^+^ (2,45 eV), V_o_^++^ (2,23 eV), O_i_ (2 eV)^35^. Besides, in our case, the ratio I_NBE_/I_DLE_ indicates the good crystallinity of produced nanostructures. It was shown previously, that the increased concentration of oxygen sites in the ZnO leads to the variation of antibacterial properties of produced nanoparticles^37,38^. Therefore, it is expectable that the different ZnO nanostructures would demonstrate various antibacterial behaviours.

The OD measurement is mainly used as a quick and affordable method to monitor the growth of bacteria during their culture in liquid media but can also be applied for testing antibacterial properties of different nanostructures and nanomaterials^39–41^. The higher the number of bacteria in the solution, the greater the OD_570_ value, and thus the lower antibacterial activity of the added material, ZnO nano- and microparticles in this case.

The high antibacterial activity towards *E. coli* bacteria showed ZnO NPs, NRs, and HSs (Fig. 5). This could be related to the specific surface area, which is the largest for 3D heterostructures, and volume to specific surface ratio, the highest for nanoparticles and nanorods, respectively. The lowest specific surface area of ZnO Ps and TPs led to a decrease in antibacterial activity. Even at the highest concentration (1 mg/mL), their viability reached about 62% and 76%, respectively.

**Figure 9:**
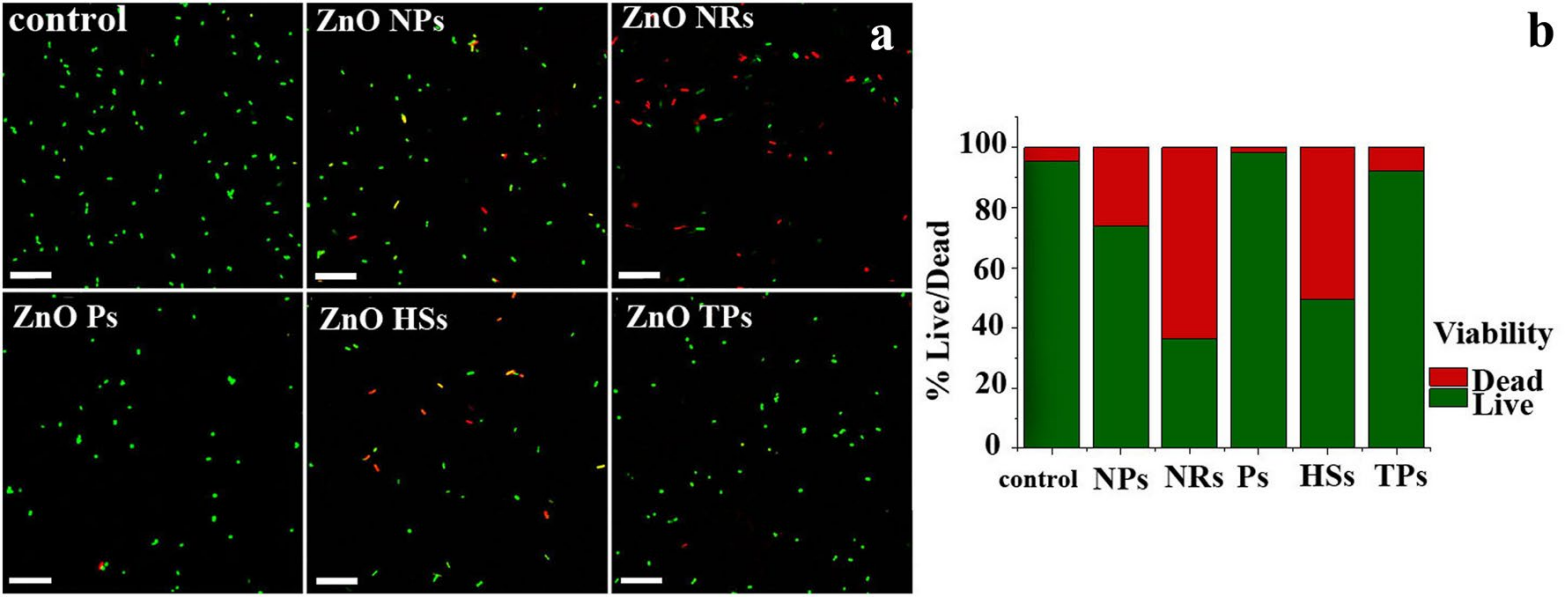
BAC Live/dead assay. (**a**) The CLSM images of *E. coli* bacteria (AATC: 35,218 *E. coli* strain) after 2 h incubation with ZnO nano- and microparticles on distinct morphology at concentration 500 μg/mL. Scale bar: 20 μm. (**b**) Relative percentage of live and dead cells in sample exposed to ZnO solutions and non-treated control as quantified from five randlomly selected microscopic images.

**Figure 10:**
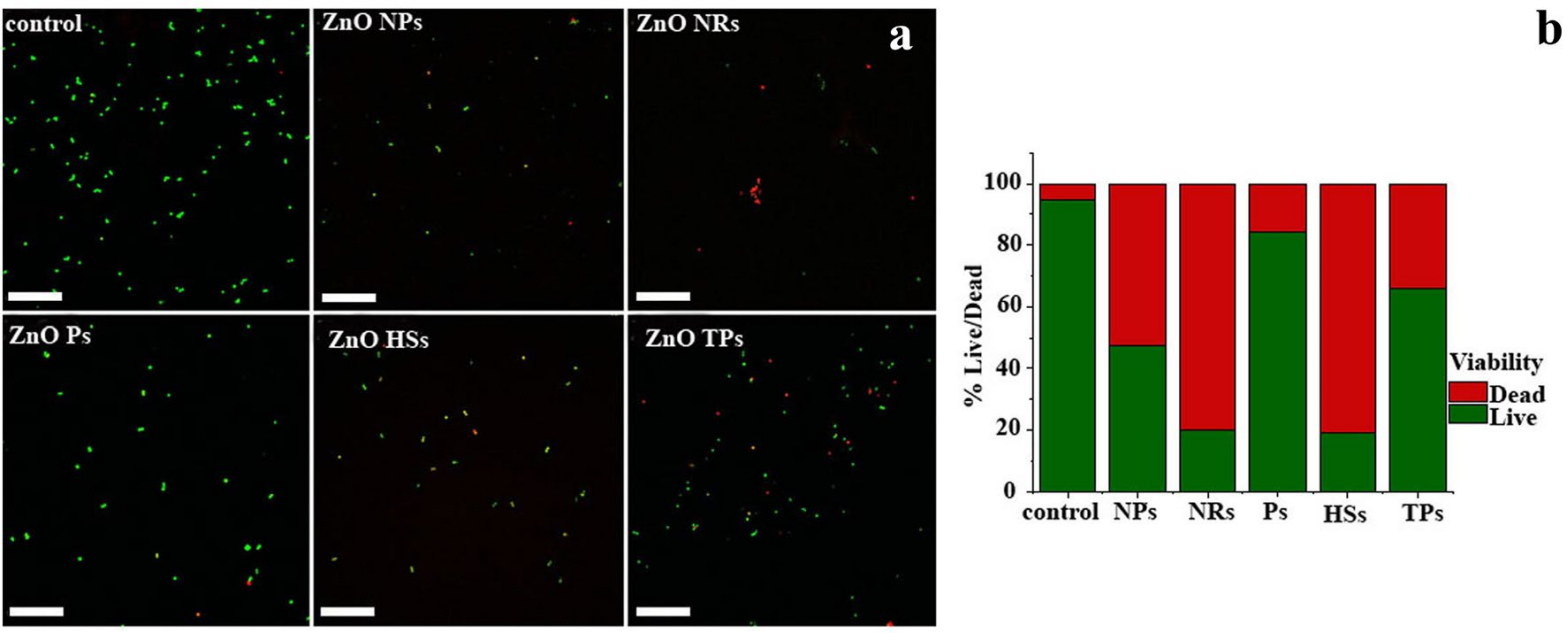
BAC Live/dead assay. (**a**) The CLSM images of *S. aureus* bacteria (AATC: 29,213 *S. aureus* strain) after 2 h incubation with ZnO nano- and microparticles on distinct morphology at concentration 500 μg/mL. Scale bar: 10 μm. (**b**) relative percentage of live and dead cells in sample exposed to ZnO solutions and non-treated control as quantified from five randomly selected microscopic images.

As it was mentioned above, the OD_570_ is proportional to the total number of bacteria, however, it does not provide any information regarding their viability. Thus, additionally, the LIVE/DEAD BacLight staining with the use of confocal laser scanning microscopy was carried out. To recognize live and dead bacteria two fluorescence dyes were used. SYTO 9 stains in green both live and dead cells, and propidium iodide (PI) stains in red dead cells, that have lost membrane integrity. Confocal images of *E. coli* and *S. aureus* were shown in Figs. 9 and 10, respectively. All of the untreated bacterial cells showed green fluorescence, due to the viable cells, indicating intact cell wall structure.

As can be seen in Fig. 9, the co-incubation of *E. coli* cells with ZnO nano- and microparticles for 2 h was enough to influence the bacteria viability. In all cases, the number of bacterial cells decreased. Moreover, the red signals, indicated dead cells appeared. The counting of both signals, collected from nine randomly selected images for all samples, allowed us to determine the percentage of live and dead cells. Based on this, we can conclude that ZnO NRs and ZnO HSs were the most effective towards *E. coli*, with the number of dead cells above 50%. As it turned out after optical density measurements, also here, the least effective were ZnO Ps and TPs.

For the *S. aureus* strain (Fig. 10) ZnO materials exhibited a stronger antibacterial effect than for *E. coli.* The number of bacterial cells was significantly reduced compared to the non-treated control. Moreover, after ZnO nanoparticles treatment the number of dead cells was about 50:50 to living cells, whereas, for ZnO NRs and HSs, the percentage of viable cells decreased to about 20%. Also, the ZnO TPs decreased cells viability, however in this case the general number of living cells seems to be higher. As in the case of *E. coli*, the ZnO particles show the weakest antibacterial activity. The obtained results for viability analysis were compatible and comparable with optical density measurements. As it was mentioned before the antibacterial effect could be related to the specific surface-to-volume ratio of ZnO materials, which is consistent with Azam et al*.*^42^ whose indicated that the antimicrobial activity increased due to a decrease in particle size of zinc oxide nanoparticles, as well as with Yamamoto^43^, who indicated that smaller size of zinc oxide nanoparticles exhibits greater antibacterial activity than microscale particles.

Four mechanisms of action have been proposed as responsible for the antibacterial properties of zinc oxide particles, namely the production of reactive oxygen species (ROS)^44,45^, the loss of cellular integrity after contact of ZnO materials and the cell wall^46^, ZnO NPs internalization^47^, as well as the release of Zn^2+^ ions^48,49^. The mechanism of nanomaterials toxicity is not specific, and thus bacteria are not able to get the resistance for nanoparticles treatment^50^. The differences in antimicrobial activity of ZnO materials used in this study depending on their size and shape could be related to the distinct mechanism of action. The smallest nanoparticles and nanorods probably internalize bacterial cells, whereas particles of micrometer size like tetrapods and heterostructures can interact with cell walls, through ion diffusion and free radicals generation, which further enter the cells, destroying cellular components such as DNA, proteins, and lipids.

Our results indicated increasing of interacellular Reactive Oxygen Species (ROS) generation in *S. aureus* bacteria cells incubated with ZnO NPs, NRs and HSs comparing to non-treated control, which is strongly related to their antimicrobial activity. Interestingly the increase of intracellular ROS in *E. coli* was observed only for heterostructures-treated cells. The lower antimicrobial activity of ZnO solutions on this strain may be related to the biological barrier for nanoparticles to pass through the *E. coli* cell wall. On the other hand, the Zn^2+^ ions, could penetrate easily through negatively charged Gram-negative bacteria cell walls^51^, which is the case with ZnO HSs that release zinc ions the most efficiently and thus exhibited high antibacterial properties.

Generally is it thought that Gram-negative bacteria are more susceptible than Gram-positive to attack by external factors, such as metal nanoparticles like it was observed for silver^52^ and gold nanoparticles^51^. As the main reason for differences in bacterial susceptibility and resistance the bacterial cell walls composition is suggested. In the case of Gram-negative bacteria, bacterial cells are covered by a layer of lipopolysaccharides (1–3 µm thick) and thin peptidoglycans (~ 8 nm thick), whereas Gram-positive bacteria possess a peptidoglycan layer (~ 80 nm thick) with covalently attached teichoic and teichuronic acids^53^. However, here we observed that Gram-negative *Escherichia coli* were less susceptible to ZnO materials than Gram-positive *S. aureus*, which is consistent with Tayel and co-workers^54^, who showed that the inhibition of Gram-negative bacteria requires higher concentrations of ZnO NPs. This is likely because the peptidoglycan layer that surrounds Gram-positive bacteria can promote ZnO attack inside the cell, while the cell wall components of Gram-negative bacteria, such as lipopolysaccharides, can counter this attack. Similar results were found by d’Agua et al*.*^55^, who showed that Gram-positive bacteria were more sensitive to peroxide hydrogen than Gram-negative bacteria. It was also seen in our earlier studies with gelatin-ZnO nanofibers^56^.

At cytotoxicity studies (Fig. 7), results showed that all nano- and microparticles are biocompatible at low concentrations (below 100 µg/mL). The greatest decrease in viability above 100 µg/mL was seen with the administration of ZnO NRs and NPs, and then HSs. The differences could be related to the distinct mechanism of action, and different levels of nanoparticles internalization. The toxicity mechanism is comparable with antibacterial action, which means that the ROS generation, mechanical harm due to direct interaction of ZnO materials with the cells, cells internalization, as well as zinc ions release could be responsible for cytotoxic activity towards human cells. Moreover, Cho et al*.*^57^ indicated that zinc oxide nanoparticles rapidly dissolve under acidic conditions (pH 4.5), which may occur after absorption of nanoparticles into lysosomes in the process of endocytosis, leading in turn to cell death.

## Conclusion

The simple chemical and physical methods were used to obtained ZnO nano- and microparticles with different morphologies. The morphology effect on luminescent and surface properties, antibacterial activity toward Gram-positive (*Staphyloccocus aureus*) and Gram-negative (*Escherichia coli*) bacteria, and cytotoxicity toward normal and cancer cells was studied. All obtained ZnO materials were highly crystalline with hexagonal wurtzite crystal structures and monodispersed. N_2_ adsorption–desorption analysis showed that ZnO in nanorange (nanoparticles and nanorods) had a higher specific surface area. All samples performed high green emission caused by structural defects in ZnO. Due to high crystallinity ZnO tetrapods (TPs) showed the strongest photoluminescence. The antimicrobial activity measurements of ZnO nano- and microparticles indicated that for all samples, the bacteria number decreased in a concentration-dependent manner. Results showed that specific surface area has a significant impact on antibacterial performance. The high specific surface area of ZnO nanoparticles led to the most effective antibacterial activity towards *E. coli* bacterial strains. Among used bacteria, the Gram-positive *S. aureus* strains were more sensitive to the antimicrobial action of ZnO nano- and microparticles than *E. coli*. The generation of reactive oxygen species is the main mechanism of ZnO NP, NRs, and HS antibacterial activity. Whereas NRs and NPs probably interact directly with bacterial cell walls and cell membranes, in case of ZnO HSs, the Zn^2−^-induced ROS accumulation is observed. All kinds of ZnO materials remain biocompatible towards cervical cancer cell line (HeLa) and normal human fibroblasts (MSU1.1) at low concentration (below 100 µg/mL). As we can see materials of different sizes and varieties of morphology can be used in biomedical fields. According to our present results, highly luminescent and biocompatible ZnO particles in nanorange (especially ZnO NPs and NRs) after biocompatibility enhancement through surface modification are planned to be used for cancer diagnostic and therapy, together with photodynamic therapy. At the same time, the microparticles will be used for biosensors, drug adsorption, and release experiments.

## Material and methods

### Materials

Zinc acetate dihydrate (Zn(CH_3_COO)_2_ × 2H_2_O, Zn(Ac)_2_, Sigma Aldrich), sodium hydroxide (NaOH, Stanlab), methanol (MeOH, Sigma Aldrich), ethanol (EtOH, Sigma Aldrich), tri-sodium citrate dihydrate (HOC(COONa)(CH_2_COONa)_2_ × 2H_2_O, Sigma Aldrich), Zn powder (Sigma Aldrich) were all of the analytical grades and used as starting materials.

### Synthesis of ZnO nano- and microparticles

#### Synthesis of ZnO nanoparticles

ZnO nano- and microparticles (nanoparticles (ZnO NPs), nanorods (ZnO NRs), hierarchical flower-like structures (ZnO HSs), and tetrapods (ZnO TPs)) were synthesized and are presented in schematic illustration (Fig. S2).

The ZnO NPs and ZnO NRs were obtained by the modified low-temperature sol–gel route described in^58^. The proposed synthesis approach is facile and allows to obtaining the crystalline monodisperse ZnO NPs (Fig. S2a) at a temperature below 100 °C. In ZnO NPs synthesis Zn(Ac)_2_ was dissolved in methanol at a constant temperature of 60 °C. Then the solution of NaOH in methanol was added to Zn(Ac)_2_ solution under vigorous stirring. The complete hydrolysis of zinc acetate with the addition of NaOH in a methanol solution results in the formation of ZnO colloid (gel). After about 3 h of stirring, white ZnO nanoparticles were separated from the mother liquor, washed with methanol twice, and dried. In the synthesis of ZnO NRs, the reaction mixture (prepared in the same manner as for ZnO NPs synthesis) was concentrated 10 times by solvent evaporation. After about 12 h of stirring white precipitate was separated, washed, and dried as described above. In the case of the ZnO NPs and NRs synthesis, the mixing of the main precursors led to the fast sol formation and then to the white homogeneous colloid solution (gel) of crystalline ZnO according to reactions (Eqs. 1 and 2).

1 $$ {\text{Zn}}^{2 + } + {\text{OH}}^{ - } \to {\text{Zn}}\left( {{\text{OH}}} \right)_{2} \downarrow $$

2 $$ {\text{Zn}}\left( {{\text{OH}}} \right)_{2} \downarrow \to {\text{ZnO}} \downarrow + {\text{H}}_{2} {\text{O}} $$

ZnO particles (ZnO Ps) were obtained using ZnO NPs (obtained earlier) by their annealing at 900 °C for 2 h (Fig. S2b). The obtaining of larger ZnO nanoparticles (ZnO Ps) with sizes approximately 150 nm was performed due to Ostwald ripening, where small crystals dissolve and redeposit onto larger crystals using high temperatures^59^.

#### Synthesis of ZnO microparticles

The ZnO 3D hierarchical structures (ZnO HSs) (Fig. S2c) were prepared by a template-free solvothermal approach according to Fang et al*.*^60^. First, Zn(Ac)_2_ and sodium citrate were dissolved in H_2_O. Separately NaOH (1 g) was dispersed in EtOH at 60 °C. After complete dissolution of both precursors, NaOH solution was added dropwise to Zn^2+^ solution. The resulting mixture was agitated at room temperature for 1 h. Then, for solvothermal reaction (150 °C for 24 h) the resulting mixture was transferred into a Teflon-lined stainless steel autoclave. After cooling down final product was centrifuged, washed with H_2_O, and dried at 60 °C for 12 h before characterization. ZnO HSs were formed from ZnO seeds by their aggregation to rods, assembly to plates, and finally to the spherical framework. The formation of the hierarchical structures is due to the use of “structural director” (generally organic polar molecules). In our case, trisodium citrate dihydrate was used for this purpose.

ZnO tetrapods (ZnO TPs), (Fig. S2d) were obtained by the simple catalyst-free oxidative-metal-vapor-transport method. The method was based on thermal evaporation of Zn powder at 1000 °C for 1 h in the air^61^. Obtaining the ZnO TPs was due to the fast formation of the ZnO seeds and then the formation of legs with preferred growth direction along the *c*-axis of the hexagonal unit cell. It is generally accepted that the nucleation and growth of ZnO tetrapods are understood to occur in the vapor phase during synthesis. The growth mechanism of the tetrahedral ZnO particles can be explained by the growth model proposed by Alsultany et al.^62^ and Markushev et al.^63^. In our case, where no metal catalyst is used, the growth of ZnO TPs can be divided into several steps—nucleation and growth. Extremely high temperature leads to the reaction of the metal Zn (Zn_met_) and then Zn gas (Zn_g_) with O_2_ gas (O_2g_) to form ZnO gas seeds (ZnO_g_), (Eqs. 3 and 4). Then during constant heating, gaseous ZnO or unoxidized Zn will condense into a liquid state of Zn_l_ or ZnO_l_ (Eq. 5), and finally following O_2_ absorption leads to the crystallization of ZnO solid seeds (*ZnO*_*s*_).

3 $$ {\text{Zn}}_{{{\text{met}}}} \to {\text{Zn}}_{g} \left( {1000\,^{ \circ } {\text{C}}} \right) $$

4 $$ {\text{Zn}}_{{\text{g}}} + {\text{O}}_{{2{\text{g}}}} \to 2{\text{ZnO}}_{{\text{g}}} $$

5 $$ {\text{ZnO}}_{{\text{g}}} \left( {{\text{Zn}}_{{\text{g}}} } \right) \to {\text{ZnO}}_{{\text{l}}} \left( {{\text{Zn}}_{{\text{l}}} } \right) $$

6 $$ {\text{ZnO}}_{{\text{l}}} \left( {{\text{Zn}}_{{\text{l}}} } \right) + {\text{O}}_{{2{\text{g}}}} \to {\text{ZnO}}_{{\text{s}}} $$

According to the octa-twin nucleus model proposed by Iwanaga et al*.*^64^, the ZnO seeds have an octahedral shape. These octahedral seeds are substrates for the following growth of ZnO TPs.

### Characterization

Structural characteristics of the obtained ZnO nano- and microparticles were measured by powder X-ray diffraction (XRD). The studies were carried out on powdered samples using an Empyrean (PANalytical) diffractometer with Cu Kɑ radiation (λ = 0.154 nm), reflection-transmission spinner (sample stage), and PIXcel 3D detector, operating in the Bragg–Brentano geometry. Scans were recorded at room temperature in angles ranging from 20° to 80° (2θ) with a step size of 0.006° and continuous scan mode. The morphology of the obtained samples was studied by high-resolution transmission electron microscopy (HRTEM; JEOL ARM 200F) and scanning electron microscopy (SEM, JEOL, JSM-7001F). For the determination of the specific surface area of the samples, the N_2_ adsorption/desorption isotherms were measured at −196 °C on a Quantachrome Nova 1000 apparatus. The samples were outgassed at 150 °C for 15 h in a vacuum before the measurements. The specific surface area was determined using the BET method. The total volume of pores was calculated using the single point mode (at p/p_0_ = 0.98). The pore size distribution was determined by applying the Barrett-Joyner-Halenda (BJH) method from the desorption branch of the isotherm. The photoluminescence (PL) of the samples was measured at room temperature using Kimmon HeCd laser. The excitation wavelength was 325 nm, and the power was around 2 mW. The PL spectra were recorded in the range from 360 to 1000 nm by Ocean Optics Spectrometer QE65 pro.

### Biological characterization

#### Cell line and cell culture conditions

Human cervical cancer cell line HeLa was obtained from American Type Culture Collection (ATCC). Human fibroblast cell line MSU1.1 was obtained from Prof. C. Kieda (CBM, CNRS, Orléans, France). Cells were cultured in a complete medium Dulbecco’s Modified Eagle’s Medium (DMEM) supplemented with 10% fetal bovine serum (FBS), 100 units/ml penicillin, 100 μg/mL streptomycin, and grown at 37 °C in a humidified atmosphere containing 5% CO_2_.

#### Cytotoxicity analysis

Cervical cancer cells (HeLa) and normal fibroblasts (MSU1.1) were used for in vitro cellular toxicity studies of ZnO nano- and microparticles. Cells (1 × 10^3^ cells/well) were seeded onto 96-well plates and incubated overnight at 37 °C under a 5% CO_2_ atmosphere. The medium in the wells was then replaced with a fresh medium containing increasing concentrations of ZnO particles (from 0.1 μg/mL to 1000 μg/mL) and incubation was continued for 24 h. The medium without ZnO particles was used as a negative control. The effect of the ZnO nano- and microparticles on cell proliferation and viability was determined by WST-1 assay according to the manufacturer’s instructions. Briefly, 10 μL of WST-1 solution was added to each well and the plates were further incubated. After 2 h the absorbance was measured with a microplate reader (AnthosZenyth 340rt) at 450 nm and 650 nm as reference. The mitochondrial function and, by extension, the relative cell viability (%) related to the negative control was calculated by test sample/negative control × 100%. Data are reported as the average ± standard deviation (SD) of wells performed in quadruplicate.

#### Bacterial growth inhibition study

Stock cultures of bacterial strains *E. coli* ATCC 35218 and *S. aureus* ATCC 29213 were stored in 30% glycerol. The strains were cultured in LB Broth Lennox at 37 °C with constant agitation at 230 rpm for 24 h. The bacterial cultures were then diluted between 2.5 × 10^5^ and 5 × 10^5^ cells/mL in LB broth medium. 100 μL of the cell suspension was then added to each well of a 96-well plate. Appropriate concentrations of freshly prepared ZnO particles solutions (10, 100, 250, 500, 1000 μg/mL) were added and placed at 37 °C in an incubator. Turbidity of the suspension, as a measure of bacteria growth, was recorded spectrophotometrically at 570 nm (OD_570_) with a microplate reader (Anthos Zenyth 340rt) after 24 h. To avoid potential interference during optical measurements caused by the light scattering properties of the solutions, the same liquid medium without microorganisms, but containing the same concentration of studied samples, was used as blank controls. The positive control was bacterial cultures without ZnO particles treatment. All experiments were performed in triplicates.

#### Bacterial cells viability analysis

To visualize the effect of *S. aureus* and *E. coli* cells' interaction with ZnO particles of different morphology, the fluorescence assay LIVE/DEAD BacLight Bacterial Viability Kit (Life Technologies) was applied and observed under a confocal laser scanning microscope (Olympus, FV1000). In brief, the *S. aureus* and *E. coli* overnight cultures were used to inoculate of fresh LB medium. At the mid-log phase of bacterial growth, the ZnO nano- and microparticles solutions were added to the final concentration of 500 μg/mL and allowed to grow for 3 h. From these cultures, 1 mL of each bacterial solution was centrifuged at 5000 rpm for 10 min. The pellets were resuspended in HEPES buffer, centrifuged, and washed with HEPES buffer three times more. Finally, the pellets were resuspended in 500 μL of HEPES buffer, and the combination of fluorescent dyes SYTO9 and PI were mixed in identical volumes. 1.5 μL of their mixture was added to each bacterial suspension and incubated in dark for 15 min. Fluorescence images were taken by trapping 5 μL of stained bacterial samples mounted on glass slides with a mounting medium and cover with coverslips. For each sample, nine randomly selected images were captured by the microscope, and live/dead cells were counted to ascertain percentage viability. Data presented are live (green) and dead (red) cells as a percentage of the total cell number (live + dead).

#### In vitro dissolution tests

To analyze the release of zinc ions from the ZnO nano- and microparticles to bulk solution, the dispersions of 100 μg/mL in DMEM medium (Gibco, supplemented with 10% fetal bovine serum, FBS), and LB Broth (Lennox, Bioshop) medium respectively, were prepared. The particle dispersion was placed into a flask (20 mL) and stirred at 37 °C for 24 h. After 15 min, 30 min, 1 h, 5, and 24 h the suspension was centrifuged at 15 000 rpm for 15 min, and the supernatant was collected. Atomic absorption spectroscopy (AAS) (contrAA® 300, Analytik Jena) was used to quantify the zinc content in the supernatant. Cell culture DMEM and LB Broth media without nanoparticles were used as references.

#### Reactive oxygen species (ROS) generation assay

The generation of ROS from ZnO nano- and microparticles was measured using 2.7-dichlorofluorescein diacetate (DCFH-DA) as a fluorescent probe on bacterial cells. Bacterial cells were inoculated in fresh LB broth and incubated at 37 °C and 200 rpm for 24 h. Afterward, the bacterial cell suspensions in mid-log phase (OD_600_ = 0,3), 100 μl was transferred to each well of a 96-well plate, and 10 or 100 μg/mL of Ag-NPs was added to the wells, and the plates were incubated for 3 h. Cells were collected by centrifugation at 5 000 rpm for 10 min and then washed and resuspended in PBS. The supernatant was labeled with 10 μM DCFH-DA and incubated for 30 min at 37 °C in dark conditions. A positive control was obtained by treating the cells with 0.8 mM H_2_O_2_ and a negative control comprising cells, without treatment, were also labeled with 10 μM DCFH-DA. The fluorescence intensity was measured using a microplate reader (Synergy-2, BioTek Instruments) with excitation at 485 nm and emission at 530 nm.

### Statistical analysis

All experiments were done in triplicate, and the results were presented as mean ± standard deviation. The experimental data were analyzed by ANOVA with post-hoc Tukey HSD test. Statistical significance was marked with asterisks depending on the *p*-value: *p ≤ 0.05, **p ≤ 0.01, ***p ≤ 0.001.

## Supplementary Information

Supplementary Information.
