# Author Correction: ZnO size and shape effect on antibacterial activity and cytotoxicity profile

**DOI:** 10.1038/s41598-023-39615-3

**Published:** 2023-08-01

**Authors:** Nataliya Babayevska, Łucja Przysiecka, Igor Iatsunskyi, Grzegorz Nowaczyk, Marcin Jarek, Ewa Janiszewska, Stefan Jurga

**Affiliations:** 1 NanoBioMedical Centre, Adam Mickiewicz University in Poznan, Wszechnicy Piastowskiej 3, 61-614 Poznań, Poland; 2 Faculty of Chemistry, Adam Mickiewicz University in Poznan, Uniwersytetu Poznanskiego 8, 61-614 Poznań, Poland

Correction to: *Scientific Reports* 10.1038/s41598-022-12134-3, published online 17 May 2022

The Acknowledgements section in the original version of this Article was incomplete. It now reads:

“The authors gratefully acknowledge the financial support by the following projects: H2020-MSCA-RISE-2017, CanBioSe 778157 (II), SONATA BIS 6 National Science Centre, Poland, under research Grant Number 2016/22/E/ST3/00458 (GN, ŁP), National Centre for Research and Development under grant NanoHEART/2021 (NB, SJ), and Ministry of Education and Science SPUB—41/E-336/SPUB/SP/2019 (MJ, SJ, GN). The authors would like to thank Katarzyna Staszak from Poznan University of Technology for her help in performing Zn release studies.”

The original Article has been corrected.

