# Supplementary material for: ZnO size and shape effect on antibacterial activity and cytotoxicity profile
Source: Sci Rep. 2022 May 17;12:8148. doi: 10.1038/s41598-022-12134-3 (PMC9114415; doi:10.1038/s41598-022-12134-3)
Supplement: Supplementary file 1 — Supplementary Information. [file 41598_2022_12134_MOESM1_ESM.doc]

Supporting information

For the article “**ZnO size and shape effect on antibacterial activity and cytotoxicity profile***”* by Nataliya Babayevska, Łucja Przysiecka, Igor Iatsunskyi, Grzegorz Nowaczyk, Marcin Jarek, Ewa Janiszewska, and Stefan Jurga.


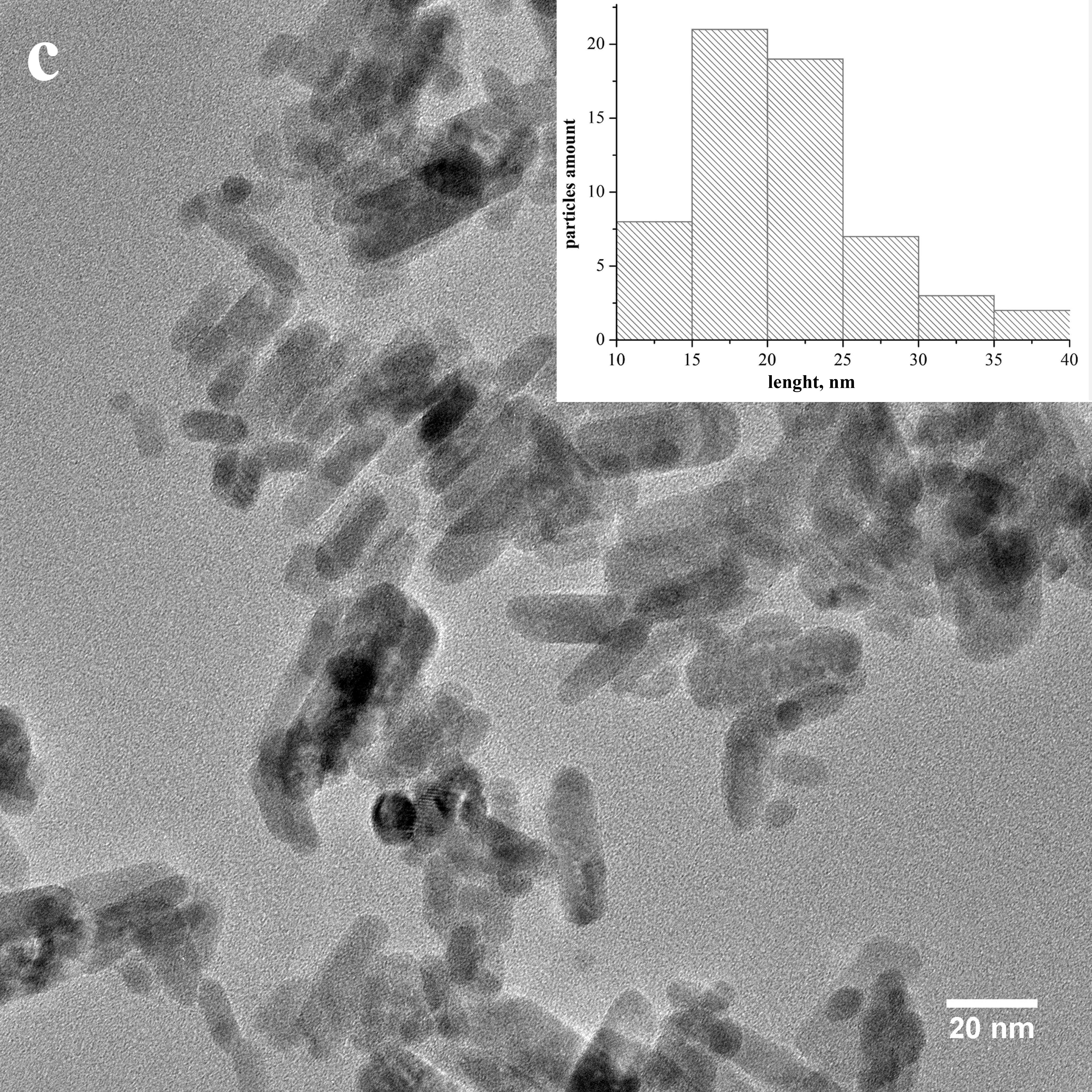


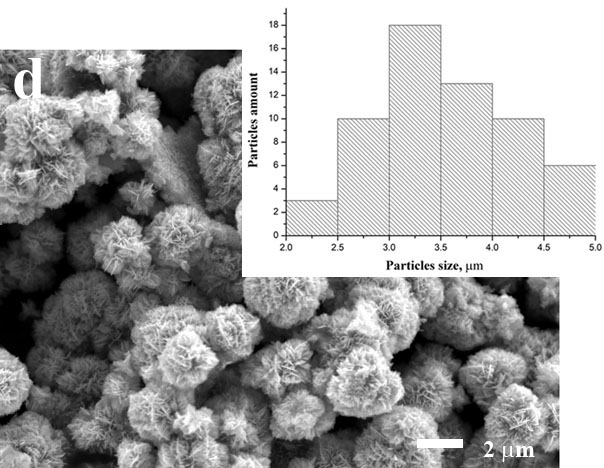

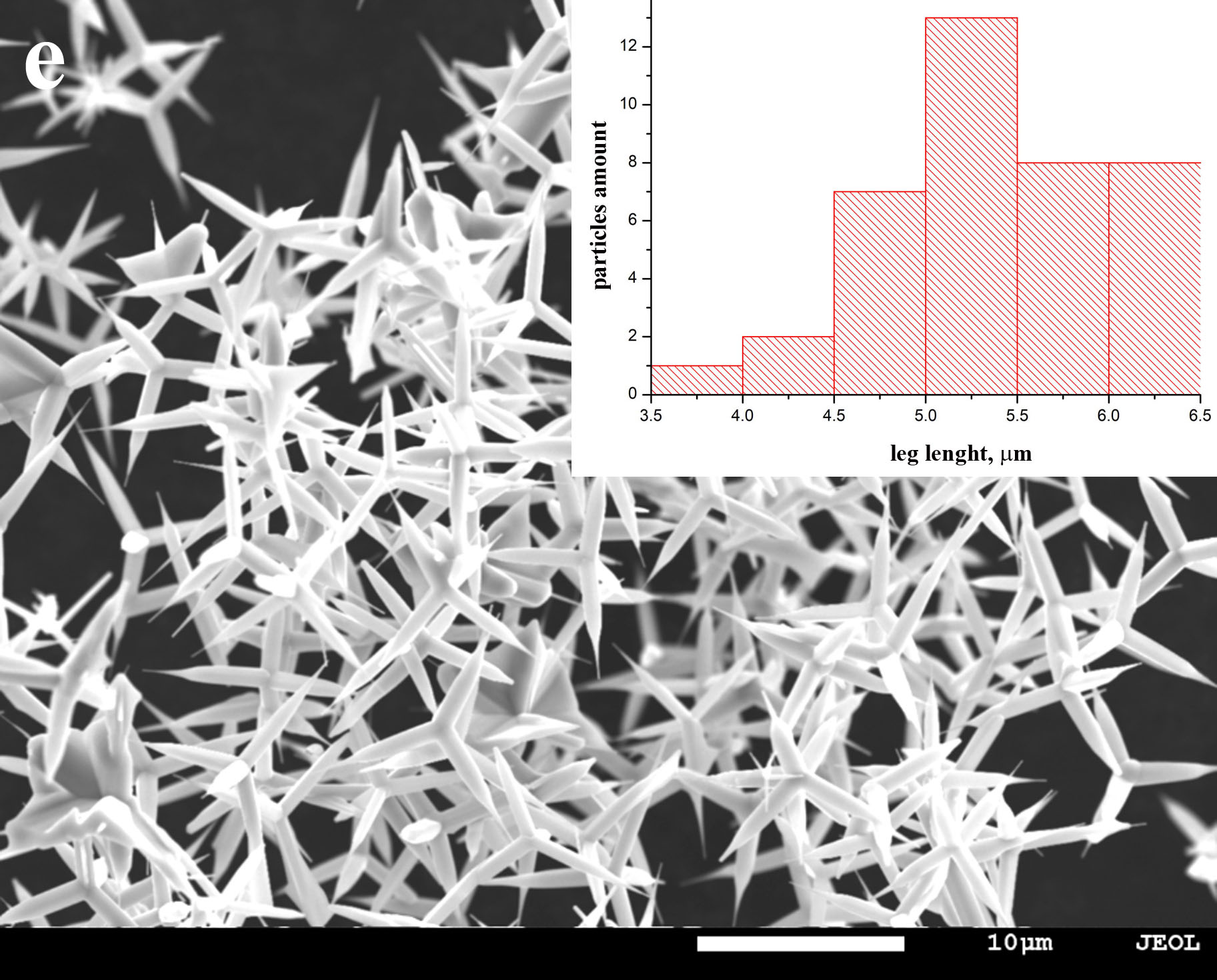


**Figure S1.** HRTEM and SEM images with histograms of the size distribution of ZnO NPs (a), individual ZnO NP (b) with of FFT (left upper corner) from the ZnO NP, ZnO NRs (c), ZnO HSs (d), and ZnO TPs (e) obtained by chemical and physical routes.


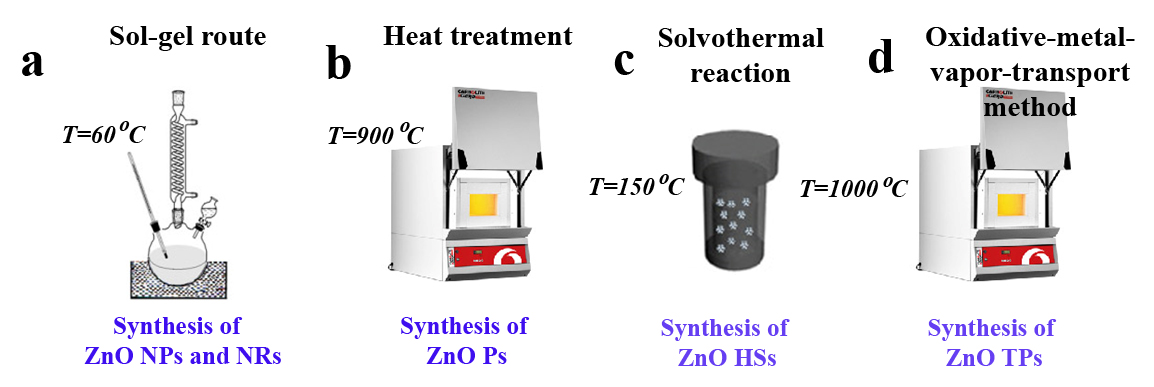


**Figure S2.** Schematic illustration of the synthesis method of ZnO nano- and microstructures.

**
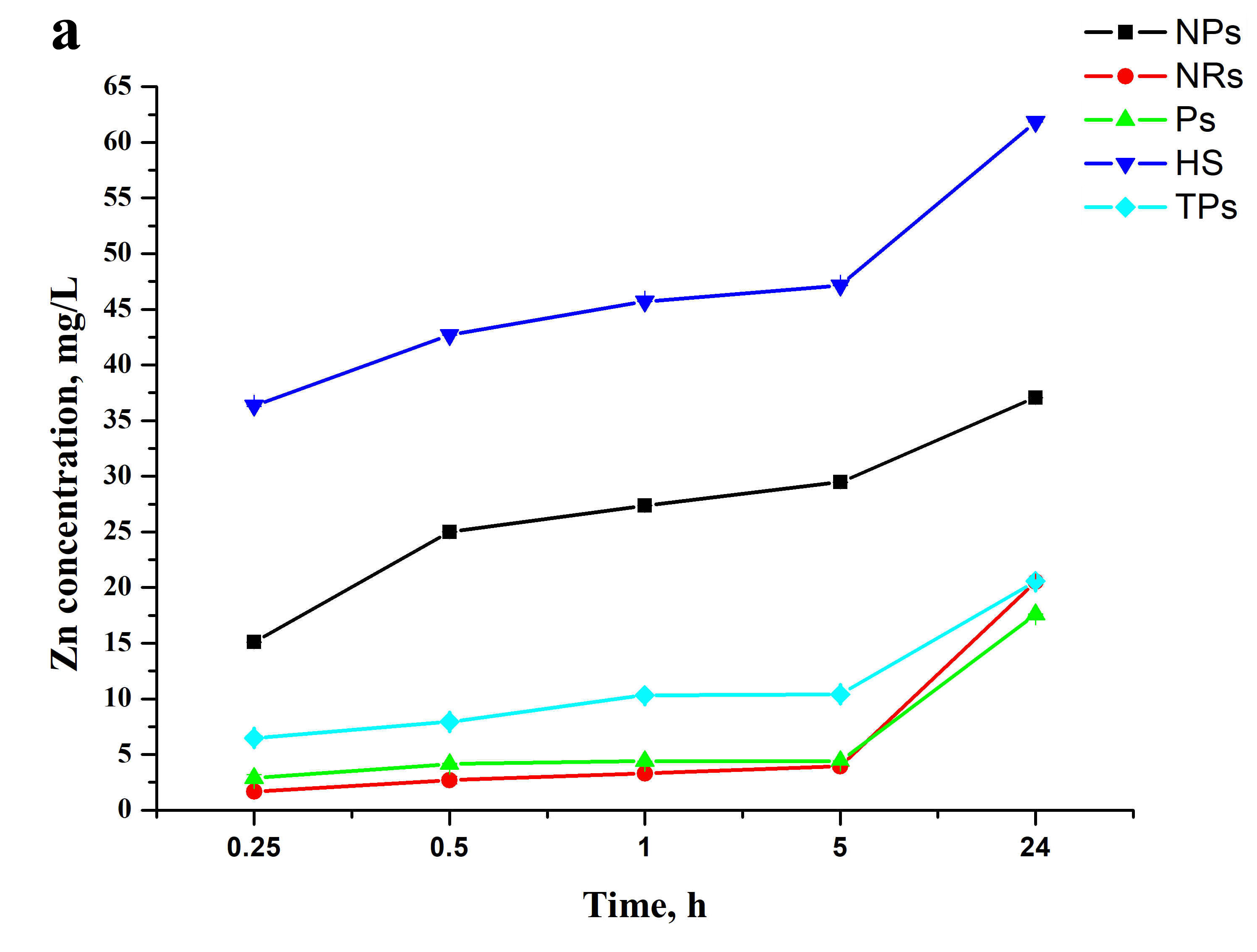
**
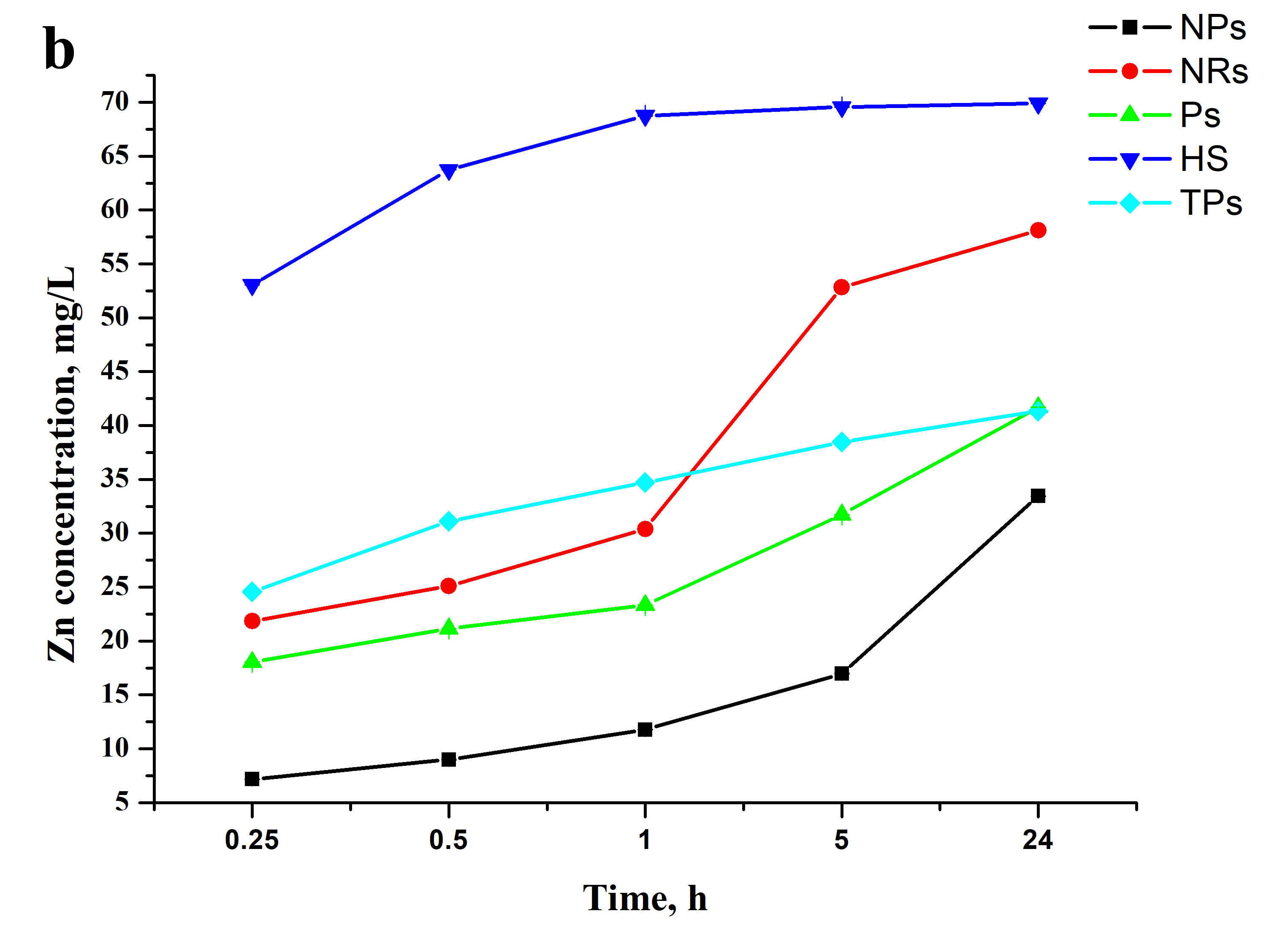


**Figure S3.** Released zinc ions in DMEM (a) and LB (b) at different times.
